# One-Year Follow-Up after Multimodal Rehabilitation for Patients with Whiplash-Associated Disorders

**DOI:** 10.3390/ijerph17134784

**Published:** 2020-07-03

**Authors:** Viktor Björsenius, Monika Löfgren, Britt-Marie Stålnacke

**Affiliations:** 1 Department of Community Medicine and Rehabilitation, Rehabilitation Medicine, Umeå University, SE-905 87 Umeå, Sweden; v.bjorsenius@gmail.com; 2 Department of Clinical Sciences, Division of Rehabilitation Medicine, Karolinska Institutet, Danderyd Hospital, SE-182 88 Stockholm, Sweden; monika.lofgren@ki.se; 3 Department of Rehabilitation Medicine, Danderyd University Hospital, SE-182 88 Stockholm, Sweden

**Keywords:** whiplash injury, pain, multimodal rehabilitation

## Abstract

Long-term symptoms after whiplash injury often comprise neck pain, headache, anxiety, depression, functional impairment and low quality of life. In an observational cohort study, we examined physical and mental health effects in patients with subacute to chronic whiplash-associated disorders (WAD) after participation in a multimodal rehabilitation (MMR) program. MMR is a team-based multi-professional method based on a bio-psycho-social model with a cognitive focus to reach an individualized and common goal for the team and patient together. Standardized self-report questionnaires were filled in three times: before MMR, after MMR, and one year after MMR. A total of 322 participants completed the program, 161 of whom responded in full and were further analyzed. At one-year follow-up after MMR, a significant improvement was seen in the evaluation of the primary outcomes (physical and mental health) and secondary outcomes (anxiety, depression, pain intensity and interference with life). Women improved on all outcomes while men did not improve on the psychological measures (mental health, depression and anxiety). This study indicates that a MMR program could be beneficial for patients with subacute to chronic WAD, at least for women, since the outcomes at one-year follow-up were positive.

## 1. Introduction

Road accidents are a common cause of traumatic neck injury/whiplash injury. The incidence of whiplash injuries in Sweden is between 1.0 and 3.2/1000 persons per year [1,2]. Whiplash trauma is most commonly associated with a motor vehicle accident, usually from the rear end or the side, which generates an acceleration/deceleration mechanism that consequently transfers energy to the neck of the driver or a passenger. This can result in a whiplash injury of bony or soft tissue which in turn can manifest clinically in various ways, such as whiplash-associated disorders (WAD), which can be classified on a five-point scale according to the Quebec Task Force Classification of Grades of Whiplash Association Disorder [3,4]. Early symptoms are neck pain, headache and dizziness [4] and additional long-term symptoms often comprise general high pain intensity, anxiety, depression, sleeping problems, functional impairment, decreased work ability and low quality of life [3,5]. Women are at a higher risk of developing such long-term symptoms [6,7,8]. It is reported that in about 50% of cases, whiplash injuries lead to chronic symptoms of neck pain one year after the injury [5,9]. However, that should be considered together with the background prevalence in systematic reviews of neck pain in the general population, where the best evidence suggests that between 20% and 40% experienced neck pain the previous month [5,10]. In Sweden, approximately 500 persons per year are unable to work because of WAD [6] which leads to a rise in medical costs with increased disability pension, lower income and income tax and generally an individual loss of well-being [3,5,11]. 

According to a report from the Swedish Whiplash Commission (2006), patients with long-term WAD should undergo rehabilitation which follows the same recommendations as other patients with long-term pain [2]. For patients with chronic pain, multimodal pain rehabilitation (MMR) is a recommended intervention. MMR is a team-based multi-professional method with coordinated measures over a limited period of time, based on a bio-psycho-social model [12] with a cognitive focus and a pedagogic approach to reach an individualized and common goal for the team and the patient together [13]. Evidence shows positive results regarding pain, function and return to work [13,14] and multiple randomized controlled studies (RCT) reveal positive results of MMR when compared with treatment as usual or waiting list controls for patients with chronic pain [14,15,16,17]. A few previous studies on patients with WAD have examined outcomes after participation in MMR [18,19] and reported positive results for pain intensity, coping ability with pain [18], physical health, working capacity, coping, vitality and medication reduction [19]. However, in those studies, follow-up was performed after just six months. Some studies have examined solely physiotherapeutic interventions in WAD patients in the context of multimodal care [20,21,22]. A systematic review reported that multimodal care that includes manual therapy, education, and exercise may benefit patients with early or persistent symptoms, however one multimodal care package was not superior to another [22]. Moreover, in a one-year follow-up study of patients with persisting symptoms within the first six weeks of injury, better results were shown for neck disability if they were given a physiotherapy package (six sessions) compared with regular advice [20]. 

There is a lack of knowledge on what the long-term effects are for patients with WAD after they have undergone MMR programs where multiple disciplines are included and where a bio-psycho-social model is in focus. In addition, it is of interest to investigate potential gender differences in rehabilitation, not least because there is an overrepresentation of women with conditions of chronic WAD and there is also a knowledge gap regarding gender-related differences on the effects of MMR. Thus, the aim of this study was to evaluate the long-term effects of MMR for patients with WAD at one-year follow-up, regarding Health Related Quality of Life, anxiety and depression, pain intensity and interference with activity. Another aim was to investigate whether there were any differences between men and women regarding the results after MMR.

## 2. Material and Methods

### 2.1. Design

This was an observational cohort study on patients with WAD with a one-year follow-up. 

Karolinska Institutet, Department of Clinical Sciences, Division of Rehabilitation Medicine, Danderyd Hospital, Stockholm, Department of Rehabilitation Medicine, Danderyd University Hospital.

### 2.2. Patients

Data were based on patients participating in an MMR program between 2009 and 2015. All patients filled in questionnaires from the Swedish Quality Registry for Pain Rehabilitation (SQRP) [23]. The study was conducted at a specialized pain rehabilitation clinic at the Department of Rehabilitation Medicine, Danderyd University Hospital, Stockholm, Sweden. All the patients suffered from pain and functional impairment after experiencing whiplash trauma which, for the majority of the patients, had occurred two to four months prior to the rehabilitation commencing.

Inclusion criteria for the MMR program and this study were (i) WAD grade I (neck pain, stiffness, no physical signs) or WAD grade II (neck complaint, musculoskeletal signs) according to the Quebec Task Force Classification of Grades of Whiplash Association Disorder [4]; (ii) age between 18 and 65 years; (iii) disabling chronic pain (i.e., on sick leave or experiencing major interference in daily life due to chronic pain); (iv) no further medical investigation needed; (v) written consent to participate in and attend the MMR program; (vi) agreement not to participate in other parallel treatments. The exclusion criteria were (i) ongoing major somatic or psychiatric disease; (ii) history of significant substance abuse; (iii) state of acute crisis. 

### 2.3. Multimodal Rehabilitation Program, MMR

In the majority of cases, the patients were referred by their primary care physician to the Department of Rehabilitation Medicine, Danderyd University Hospital. After referral, a nurse made a primary assessment of the patient’s life situation and started the process of setting goals and informing the patient about the program. The patients were assessed by multi-professional teams (a specialist physician in pain and rehabilitation medicine, a psychologist, an occupational therapist, a physiotherapist, a social worker and a nurse) before their participation in MMR. The physician’s, assessment included the following: a pain history and clinical examination to exclude serious underlying conditions, referred neck pain; need of further investigations, e.g., imaging, blood tests, other specialist referrals; and/or optimization of the pharmacological treatment. If the patient decided to take part in the program and if the inclusion criteria were fulfilled, the patient and the other team members assembled to do a more in-depth assessment of the patient’s life situation. The disciplines included in the program were the same as in the assessment. All the disciplines were given more or less the same amount of time. All had comprehensive knowledge and experience of WAD-related rehabilitation. The duration of the program was five weeks with a total of 17 sessions, each lasting for 1.5 h with 1–3 sessions a day, 2–3 days a week. The patients were in groups of eight, they participated in all sessions and peer learning was facilitated by two patients being admitted and two patients discharged every week. 

One of the tasks of the nurse and the physiotherapist was to inform the patients about symptoms related to WAD, pain physiology, mechanisms related to the injury and healing, pain medication, pain management, the relationship between pain, bodily signs, behavior and emotions, and, together with the social worker, to give information on social rights and insurance. A group discussion based on a social and psychological perspective was conducted with the social worker and the psychologist. If needed, individual meetings were arranged with the psychologist. In addition, the physiotherapist handled sessions of 1.5 h per week on body awareness, relaxation and physical activity, and patients could also get a prescription for a transcutaneous electrical nerve stimulation device (TENS). The occupational therapist held sessions of 1.5 h per week on ergonomics regarding sleep, work and daily activities, how to balance activity and rest, and EMG bio-feedback. There were also 30-minute scheduled walks every week with a focus on mindfulness. 

The aim of the program was to help the patients reach an acceptance of their situation and to encourage a successive return to activities and participation, all through early rehabilitation. At the end of the MMR program, a plan was set up by the patient and the physician together for how the patient would be able to return to work. The physiotherapist gave individualized recommendations on physical activities and a nurse ended the program with a final visit with a discussion on further self-management strategies for optimal individual rehabilitation.

### 2.4. Measures

Patients filled in questionnaires from the SQRP [23] before MMR (baseline measures), immediately after MMR and at one-year follow-up after MMR. The baseline questionnaire also included questions about gender, place of birth, job status, expectations of recovery and return to work/extend work hours. The first two questionnaires were completed at the clinic while the follow-up was done by email. The measures used are recommended in national and international guidelines to describe the health status of patients with chronic pain and to follow up on results after rehabilitation. 

#### 2.4.1. The Short Form Health Survey (SF36) Standard Swedish Version 1.0

SF36 [24,25] is used to measure physical and mental health, functional level and pain and its influence on quality of life and their changes over a period of time. The instrument comprises eight subscales and is used to measure the effect of a disease or treatment and provide a basis for an index value for a Physical Component Summary (PCS SF36) and a Mental Component Summary (MCS SF36). Both of these indexes were used as primary outcomes in this study. Norm values for the general Swedish population are PCS 50.0 SD 9.7 (6.8–73.7), MCS 50.0 SD 10.3 (49.8–50.2) [25]

#### 2.4.2. The Hospital Anxiety and Depression Scale (HAD) 

HAD [26] is a questionnaire for the estimation of anxiety and depression. It includes fourteen questions: seven questions for each subscale which are scored separately. HAD does not provide any specific diagnostic conclusions but is a tool to detect patients who are at elevated risk for disorders of anxiety and depression and to assess symptom severity. Cut-off scores are available for the quantification of each sub-scale of anxiety and depression: 0–7 points indicates low risk, 8–10 points indicates a risk, 11–21 points indicates a likely risk (further 11–14 indicates moderate risk and 14–21 indicates a severe risk). 

#### 2.4.3. Numeric Rating Scale (NRS)

NRS [27] is a measure for estimating pain intensity the previous week. It is an 11-point numeric scale from 0, representing no pain, to 10, representing the worst imaginable pain. Clinical significant 

Changes are: 10–20% minimal important, ≥30% moderately, ≥50% substantial.

#### 2.4.4. Multidimensional Pain Inventory (MPI) 

The MPI [28] is a self-reporting questionnaire of 61 questions to assess medical, psychosocial and behavioral experiences. It consists of three sections and in this study, we used a subscale for interference with life. MPI-Interference. It is a validated 7-pointed numeric scale that measures the patient’s perception of to what extent pain interferes with life, such as marital and family functioning, daily social activities and work and how satisfied the patient is with their current level of functioning. Higher score indicates higher impact on interference in daily life. 

### 2.5. Data Analysis

An assessment of data was performed before the MMR commenced, immediately after and one year after the completed MMR. Statistical Packages for the Social Sciences, version 22.0, (IBM Corp., Armonk, NY, USA), were used for data preparation and statistical analysis. Descriptive statistics were used to describe the population of the study. Data are reported as means with SD and medians. A chi-square test and Fischer´s exact test were used for frequency data. The Mann–Whitney U-test was used to compare the different groups´ demographic data and outcome scores at baseline. The Friedman test was used for analyses over time and the Wilcoxon signed ranks test was used for post-hoc analysis. The significance level was set at 0.05.

### 2.6. Ethics

This study was conducted in accordance with the ethical principles of the World Medical Association Declaration of Helsinki. In accordance with the routines of the SQRP, all subjects received written information that the questionnaires might be used for research purposes and that participation was entirely voluntary. Informed consent was received from all participants. The data were collected as part of the ongoing quality management of clinical care activities in the participating departments, and the data were stored with the consent of the National Swedish Data Inspection Agency (permission no. 1580-97). All possible identifications were deleted before statistical analysis.

## 3. Results

### 3.1. Patients

**Figure 1:**
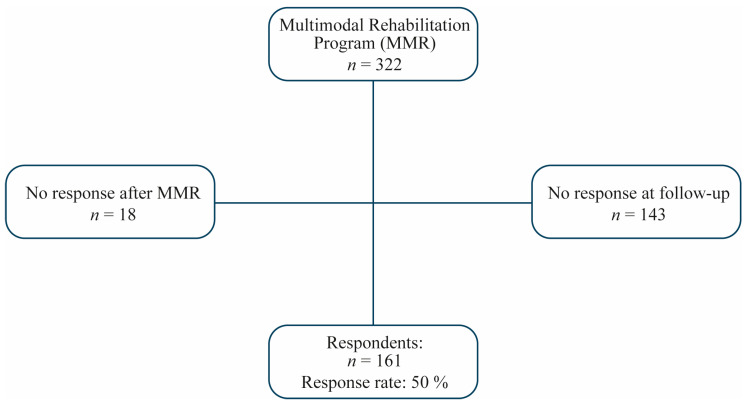
Flow chart. Self-completed questionnaires were given to the patients for them to fill in before, immediately after, and at one-year follow-up after multimodal rehabilitation (MMR). Measures of outcome were: The Short Form health survey (SF-36), Hospital Anxiety and Depression scale (HAD), Numeric Rating Scale (NRS) and interference subscale of Multidimensional Pain Inventory (MPI).

A total number of 322 participants were enrolled in the MMR program. Patients who failed to complete one or more of the three questionnaires were excluded. An amount of 18 patients did not answer after MMR and a further 143 patients did not answer at follow-up. The total number of non-respondents was 161 patients as illustrated in Figure 1. 

### 3.2. Analysis of Non-Respondents 

We compared patients who had completed all three questionnaires, denoted as respondents (*n* = 161), with excluded patients, or non-respondents (*n* = 161). There was a significant difference in gender with a higher proportion of men among non-respondents (58.4%) than in the group of respondents (41.6%) (*p* = 0.022), At baseline, the non-respondents had significantly higher depression scores (*p* = 0.016), and anxiety scores on the HAD (*p* = 0.044)) and a significantly lower rate in physical health on the SF-36 (*p* = 0.011) than the group of full respondents. No significant differences existed between the groups regarding age, country of birth, job status, pain duration, conviction of recovery and expectations on when and how return-to-work would be. 

### 3.3. Patient Characteristics for the Analyzed Group

Background data and characteristics of pain are presented in Table 1 and Table 2. More than two-thirds of the patients were women (68%). There was a significant difference in place of birth between the gender groups (*p* = 0.017) with a higher proportion of men born outside of Europe in comparison with women. Regarding educational level, 21.2% of the men and 38.5% of the women had completed university education. The mean number of anatomical regions affected by pain was 10.57 (SD 5.28) out of 36 pre-defined regions with no significant difference between women and men (*p* = 0.359). 

### 3.4. Physical and Mental Health (Primary Outcomes)

In total, a significant improvement was seen for the primary outcomes of SF-36 (see Table 3). For the group of all patients, both physical health and mental health on the SF-36 improved significantly from baseline to after MMR and from baseline to one-year follow-up (*p* < 0.001). Mental health improved significantly for women (*p* < 0.001) at one-year follow up, but not for men (*p* = 0.157).

### 3.5. Pain Intensity, Anxiety and Depression, Interference with Life (Secondary Outcomes)

For the group of all patients, the secondary outcomes showed a significant improvement from baseline to after MMR and from baseline to one-year follow-up (see Table 3). A total significant improvement on anxiety (*p* = 0.002) and depression (*p* = 0.044) was seen which is also reflected in a significant proportional change at follow-up compared with baseline for patients scoring moderate/severe anxiety from 78% to 38% (*p* < 0.001) and depression from 35.4% to 29.8% (*p* = 0.026) on the HAD. Anxiety in men was significantly improved after MMR (*p* = 0.008) but not at one-year follow-up (*p* = 0.118), whereas women improved significantly only after one year (*p* = 0.002). Regarding pain intensity on the NRS, there was a significant reduction after MMR and at one-year follow-up (*p* < 0.001) in all patients. The results for MPI-interference with activity showed a significant improvement for all patients both from baseline to after MMR (*p* < 0.001) and from baseline to one-year follow-up (*p* < 0.001). 

## 4. Discussion

This study shows positive effects in patients with subacute to chronic WAD with regard to physical and mental health, anxiety and depression, pain intensity and activity after participation in a five-week outpatient MMR program and at one-year follow-up. The findings indicate a potential effectiveness of MMR in a long-term perspective for patients with WAD, with a greater improvement of mental health and depression in women. While women showed significant positive results for these psychological factors, for men, some of these aspects worsened. Pain intensity and activity were significantly improved in all the received questionnaires.

A detailed analysis of the results in our study reveals that a significant improvement was seen in physical health, both after MMR and at one-year follow-up. This is in line with results from some earlier studies. However, our results reveal persisting positive results for a longer time perspective than that in other studies. A comparable rehabilitation program to ours was examined in a study by Angst et al. [19] on WAD patients with a six-months follow-up. That study showed positive results and a moderate effect size (ES) on physical health on the PCS-SF36 (*p* <0.001, ES 0.71). In that study, no observation was carried out on gender groups specifically. Although the program studied was similar to ours regarding content and number of professions, the patients were enrolled on the program at a later stage after trauma (“time since trauma”: 10 months) than the patients in our study (“mean duration of pain”: three months). Also, some other previously evaluated MMR programs for WAD patients included only one discipline, such as a physiotherapist [20,21,22] or a general practitioner [29] and some did not include psychological interventions [21,29]. In a study by Lamb et al. [20], a physiotherapy package (up to six treatments) was compared with a single physiotherapy session. They found no short or long-term (one year) beneficial significance with regard to the SF-12 subscales for physical and mental health-related quality of life (HRQoL). This might indicate that an MMR program with several disciplines and a rather intensive model of treatment is a beneficial factor for achieving improved physical health in WAD patients and is in line with previous research with evidence that favors MMR compared to less comprehensive treatments for patients with chronic pain [14,15,16,17]

Subacute to chronic WAD is a heterogenous condition that includes a complex interaction of psychological and physical factors. The population of all patients in the present study showed a significant improvement at follow-up for psychological factors of mental health, anxiety and depression. Conflicting results to this were seen in a previous study of patients with WAD participating in a rehabilitation program where no significant improvement in these variables were seen at follow-up after six months [19]. However, the patients in our study were enrolled at an earlier stage after the trauma which possibly favors a more successful rehabilitation. Additionally, it could be speculatively assumed that the system used in the MMR program of our study, whereby two patients were admitted and two were discharged every week, might contribute to the psychological progress within the group and among its members. Patients who were at a later stage of the program had often built up an understanding, perhaps an acceptance of their pain and the situation they were in and were therefore able to exert a positive influence on newcomers to the group. Patients were referred to the pain rehabilitation clinic because of WAD, including chronic pain. It is well known that chronic pain can trigger depressive symptoms and that depression in turn increases the adverse effects of pain. These two conditions work in a negatively synergic way in that the depression reinforces the chronic pain and the chronic pain promotes depressive symptoms which altogether can manifest in lower levels of activity and quality of life. It seems that our MMR cognitive-behavioral program may have affected the group of all patients’ self-perceived anxiety and depression levels

Differences between men and women were found regarding psychological measures. Women showed significant improvement regarding mental health and depression at one-year follow-up, while the corresponding improvement was not shown in men. Instead, the score of depression in men slightly increased at one-year follow-up, with a significant change in anxiety after MMR, but not at one-year follow-up. These findings indicate that men did not maintain the psychological improvement that was shown after MMR; instead, there was a tendency that they were slightly more depressed at follow-up. An earlier study investigating gender differences in chronic WAD patients showed small differences in men and women regarding psychosocial factors but found that women reported significantly higher social companionship and emotional support in problem situations [7]. It is possible that a difference in educational level between the gender groups had an impact, with a smaller proportion of men who had completed university education compared with women. These might be favorable factors for women’s rehabilitation and might explain the gender differences observed. 

The interference with activity was significantly reduced in both gender groups at one-year follow-up. A previous study on patients with disabling pain who underwent an MMR program (*n* = 1468) showed significantly reduced interference after discharge and at one-year follow-up [30]. This is in line with our results, but in contrast to a study by Sterner et al. (2001) where WAD patients showed worsened score of activity at a six-month follow-up after an early interdisciplinary rehabilitation program [18]. However, patients in this study had a longer post-trauma medium inclusion than the subjects in our study (eight months compared with approximately three months) which might explain the difference in outcome and the need to offer a rehabilitation intervention earlier after the trauma. 

In our study, pain intensity was significantly reduced for all patients, in line with some previous studies of MMR on patients with WAD, Sterner et al. (2001) reported decreased pain intensity, coping ability with pain and control of it at six-month follow-up [18]. Similar results on pain, measured with the North American Spine Society cervical spine self-assessment instrument (NASS), were seen in another six-month follow-up (*p* = 0.001) in a study by Angst et al. [19]. Thus, there is conformity with previous results for a time duration of six months. Although our study reveals consistent positive results after 12 months, pain intensity was high at all time points with a minimal important clinical change on the NRS. A difference between gender groups was seen with a non-significant but slight increase of median pain intensity in men at follow-up (6.0) compared with immediately after MMR (5.0). There seems to be a pattern in our results indicating that MMR was more successful for women than men.

There is an overrepresentation of women with conditions of chronic WAD and women with chronic pain are overrepresented in specialist clinics in Sweden compared to the community prevalence [23]. More than twice as many women than men were enrolled for MMR in our study, which was also the case with many previous studies [13]. The greater number of women in MMR might be explained by women´s increased susceptibility for many chronic pain conditions [31] as well as for developing chronic WAD [8]. Bearing this in mind, our results of better long-term improvement in women regarding mental health and depression are of significant interest. It could be thought that external factors linked to the MMR program, such as social and emotional support, are positive for women´s rehabilitation in a group where women are in a substantial majority, as in this studied group. It is possible that certain interventions in our MMR program contribute to the positive short and long-term results shown and the gender differences observed. Women’s higher education level (although non-significant) may have contributed to their benefit from the cognitive-behavioral-based MMR-program. However, this cannot be fully concluded and it cannot be excluded that a spontaneous improvement occurred since no comparison with a control group was made. 

### Strengths and Limitations

One of the strengths of our study is the self-assessments, which are included in the SQRP and are reliable and validated measures for WAD patients [23,32]. The data make up a relatively large sample size, which gives clinical relevance, as the collection was carried out in clinical practice. Additionally, since it was a register study based on the SQRP, the collection of data was carried out in a correct and standardized way. Another strength is the clinical and observational setting of the study which allowed a naturalistic investigation without interfering with or influencing the process of the intervention. General practitioners referred the patients to a specialist pain rehabilitation clinic because of subacute to chronic WAD, thus, the patients represent a selected group with more severe or complex consequences of pain than patients being treated in primary care

The main limitation of the study is that no randomized control group was used and thus it is hard to eradicate conclusions of random effects on the course of treatment effects. Although the sample of patients were representative for participants in MMR during 2009–2015, another limitation is the high rate of non-respondents, 161/322 (50%) with 58.4 % of all the male respondents being in this group. The considerable majority did not complete at follow-up and this may have affected the results. A significantly lower score of physical health and more anxiety and depression was seen in this group which could explain their reason for not completing the questionnaire. A problem to be considered, which is also pointed out by Hurwitz et al. (2009), is the risk of biased conclusions on the effectiveness of MMR on patients with WAD and their specific interventions as it is difficult to extract results of certain interventions from other interventions embedded in multimodal packages combined in previous studies reviews [33]. In addition, since the long-time variables were investigated one year after MMR one cannot rule out that systematic distortions as well as other factors and interventions might have influenced the results. The study used validated instruments from SQRP, but no specific instrument for WAD was included. 

## 5. Conclusions

This study has implications for clinicians. Due to the complexity of WAD, the present study indicates that a five-week MMR program could be beneficial for patients with subacute to chronic WAD on physical and mental health, anxiety and depression, pain intensity and activity. Further controlled studies are needed to provide better knowledge of the long-term effects of MMR in WAD patients with a design that enables a more precise observation of what favorable factors there are in MMR, both in general and for each gender group. Specific instruments for WAD such as the Neck Disability Index could be used. 

## Figures and Tables

**Table 1:** Patient demographics and pain characteristics. Values for women and men are reported separately.

	Total Respondents	Women	Men	*p*-Value
N	161	109	52	
Percentage frequency	100%	68%	32%	
Age (mean, SD)	39 (11.1)	38.5 (11.3)	41 (10.7)	
Highest educational level (within gender)				n.s
Primary school	8.10%	7.30%	9.60%	
Secondary school	54.70%	48.60%	67.30%	
University	32.90%	38.50%	21.20%	
Other	−	5.50%	1.90%	
Missing data	−	−	−	
Place of birth (within gender)				0.017
Sweden	59.40%	66%	44.20%	
Nordic country	1.90%	2.80%	0.00%	
Europe	5.00%	3.70%	7.70%	
Country outside Europe	33.80%	26.90%	48.10%	
Missing data	0.60%	0.60%	−	
Job status (within gender)				n.s
Employed	90.10%	88.10%	94.20%	
Unemployed	3.70%	3.70%	3.80%	
Unemployed	1.20%	1.80%	0%	
Not acquisition worker	1.20%	1.80%	0%	
Missing data	−	−	−	

n.s: non-significant.

**Table 2:** Pain characteristics. Values for women and men are reported separately.

	*n*	Total Respondents	*n*	Women	*n*	Men	*p*-Value
Pain duration (median, quartiles 25; 75)	153	91 days (51.5; 158.5)	101	97 days (51.5; 171.5)	52	90 days (52; 128)	n.s
How convinced are you about recovery?	150						n.s
1–3: Positive outlook	122	75.80%	82	75.30%	40	76.90%	
4–5: Negative outlook	28	17.40%	18	16.50%	10	19.20%	
Missing data	11	6.80%	9	8.30%	2	3.80%	
How do you think it will be to return to work, to study, or extend working hours?	93						n.s
1–3: very easy	36	38.70%	26	39.40%	10	37%	
4–5: very difficult	32	34.40%	21	31.80%	11	40.2	
Missing data	25	26.90%	19	28.80%	6	22.20%	
When do you expect to return to work, to study, or to extend your working hours?	93						n.s
1–3: as soon as possible	55	57.60%	38	57.60%	17	63%	
4–5: never	8	8.70%	5	7.60%	3	11.10%	
Missing data	30	32.30%	23	34.80%	7	25.90%	

n.s (non-significant).

**Table 3:** Course of whiplash injury patients on self-assessments (*n* = 161). Primary and secondary outcomes presented in medians and p-values. Presentation in total and for gender groups before (a), after MMR (b) and at one-year follow-up (c). * denotes significant difference (*p* < 0.05).

	*n*	Missing	Before	After	One-Year Follow-Up	*p*-Value	*p*-Value	*p*-Value
			(a)	(b)	(c)	Friedman test	(a–b)	(a–c)
PCS SF-36								
median (range)								
Women	101	8	32 (18–51)	34 (19–52)	34 (11–57)	0.007 *	0.021 *	0.003 *
Men	50	2	31 (15–54)	33 (17–54)	34.5 (15–58)	0.020 *	0.014 *	0.002 *
Total	151	10	32 (15–54)	34 (17–54)	34 (11–58)	<0.001 *	0.001 *	<0.001 *
MCS SF-36								
Women	101	8	29 (8–60)	30 (9–61)	39 (11–58)	<0.001 *	0.214	<0.001 *
Men	50	2	32 (11–52)	32 (9–61)	33 (10–59)	0.805	0.933	0.157
Total	151	10	29.5 (8–60)	30.5 (9–61)	37 (10–59)	<0.001 *	0.208	<0.001 *
HAD-A								
Median(range)								
Women	109	−	10.0 (0–21)	9.0 (0-21)	9.0 (0–21)	0.011*	0.159	0.002 *
Men	52	−	10.0 (2–18)	8.5 (1-21)	8.5 (0–21)	0.044 *	0.008 *	0.118
Total	161	−	10.0 (0–21)	9.0 (0-21)	9.0 (0–21)	0.002 *	0.011 *	0.001 *
HAD-D								
Women	109	−	8.0 (0–20)	8.0 (0–18)	6.0 (0–21)	0.004 *	0.103	0.001 *
Men	52	−	9.0 (0–19)	8.0 (0–20)	9.5 (0–20)	0.284	0.142	0.807
Total	161	−	8.0 (0–20)	8.0 (0–20)	7.0 (0–21)	0.044 *	0.029 *	0.011 *
NRS								
median (range)								
Women	102	7	7.0 (1–10)	6.0 (1–10)	6.0 (0–10)	<0.001 *	<0.001 *	<0.001 *
Men	47	5	7.0 (2–10)	5.0 (1–9)	6.0 (0–9)	<0.001 *	<0.001 *	<0.001 *
Total	149	12	7.0 (1–10)	6.0 (1–10)	6.0 (0–10)	<0.001 *	<0.001 *	<0.001 *
MPI interference								
Median (range)								
Women	108	1	4.3 (0.36–6)	4.1 (0.6–6)	3.9 (0–6)	0.001 *	0.088 *	<0.001 *
Men	52	−	4 (0.6–5.9)	4 (0.45–5.9)	3.9 (0.1–6)	0.013 *	0.019 *	0.011 *
Total	160	1	4.2 (0.7–6)	4.1 (0.45–6)	3.9 (0–6)	<0.001 *	0.004 *	<0.001 *

PCS SF36: Physical Component Summary; MCS SF36: Mental Component Summary; HAD-A: anxiety component of Hospital Anxiety and Depression scale; HAD-D: depression component of HAD; NRS: Numeric Rating Scale, ´pain average last week´; MPI: Multidimensional Pain Inventory, subscale interference.
